# Co-morbid mental disability among Chinese elderly with motor disability: Based on a nationwide survey

**DOI:** 10.1371/journal.pone.0195555

**Published:** 2018-04-05

**Authors:** Chao Guo, Ping He, Xinming Song, Gong Chen, Xiaoying Zheng

**Affiliations:** 1 Institute of Population Research, Peking University, Beijing, China; 2 APEC Health Science Academy (HeSAY), Peking University, Beijing, China; 3 China Center for Health Development Studies, Peking University, Beijing, China; Universita degli Studi di Perugia, ITALY

## Abstract

This study aimed to investigate the prevalence rate and associated factors of co-morbid mental disability among the elderly with motor disability. Data was obtained from the second China National Sample Survey on Disability (CNSSD). 250,752 older adults aged 65 years and above were sampled from 5964 sites, 2980 towns, 734 counties in 31 provinces in China. Descriptive statistics were used for population-weighted numbers and prevalence of co-morbid mental disability by different characteristics. Univariate logistic regression and multivariate logistic regression were used to calculate the association between mental disability, and the factors of co-morbid mental disability among physical disability. Among the elderly with motor disability, 275,607 persons (2.28%, 99% CI: 1.97–2.59) had co-morbid mental disability at the survey time, which was significantly higher than those without motor disability (OR: 2.11, 99% CI: 1.83–2.43). The factors associated with a higher risk of co-morbid mental disability included urban areas (OR: 1.38; 99% CI: 1.04–1.84), east (2.60, 1.76–3.83) or west (1.65, 1.06–2.55) regions, having other co-morbid disabilities (5.93, 4.48–7.85), and having more severe motor disability (Moderate: 1.81, 1.28–2.57; Severe: 2.90, 2.08–4.04; Extremely severe: 4.22, 2.96–6.01). The elderly with motor disability have a high risk of co-morbid mental disability, especially for higher socioeconomic groups. These findings highlight the need of and will be beneficial for implementing more comprehensive prevention and rehabilitation strategies, to prevent and control the emergence and development of multiple disabilities and promote the physical and mental health of the elderly.

## Introduction

Disability has become a global challenge of population health. In fact, a substantial proportion of people living with disabilities were older population across nations. Previous studies indicated that 35.6%, 39.8%, and 54.3% of people with disabilities were elderly aged 65 years and above in the United State, Canada, and Germany, respectively [1].

Previous studies indicated that in persons over the age of sixty the co-morbid presence of two or more diseases or disabilities has become the rule rather than an exception. For example, 55% of the elderly aged 75 and above suffered from more than four diseases in Netherlands [2,3]. In Canada, 90% of the elderly aged 65 and above had more than two diseases [4,5]. This means that in the years ahead multiple disabilities will be an even greater concern to developed and developing nations due to the higher risk of disability in older people and the increase of the chronic diseases prevalence.

With the development of ‘bio-psycho-social model’, mental health and physical health have been proven inextricably linked. Over the past two decades, the prevalence of co-morbid mental and various physical diseases has increased dramatically, especially in older adults [6]. Due to the concurrence of mental and motor disabilities, there are more difficulties ahead in the daily life and social participation among the elderly.

As the most populous nation, China has more than 130 million older adults aged 65 years and above, among which 38 million were diagnosed with one or more disabilities that affect their daily lives and social activities [7]. However, only a limited number of studies have reported the mental health in persons with disabilities [8] and none has estimated the co-morbid mental disability among those with motor disability in a population-based sample.

Using nationally representative population-based data, this study aimed to investigate the prevalence rate and associated factors of co-morbid mental disability among the elderly with motor disability.

## Materials and methods

### Data source

Data were obtained from the second China National Sample Survey on Disability (CNSSD), a nationally representative survey aiming to investigate the prevalence, causes, and severity of disabilities in China. The survey was approved by the State Council of China (No. 20051104) and conducted according to legal guidelines governed by the Statistical Law of the People’s Republic of China (1996 Amendment) [8]. All respondents provided consent to participate in the survey and to undergo clinical examinations for diagnoses. Experts from the National Bureau of Statistics of China, the China Federation of Disabled Persons, and the Division of Statistics of the United Nations reviewed the survey protocol and questions.

**Fig 1:**
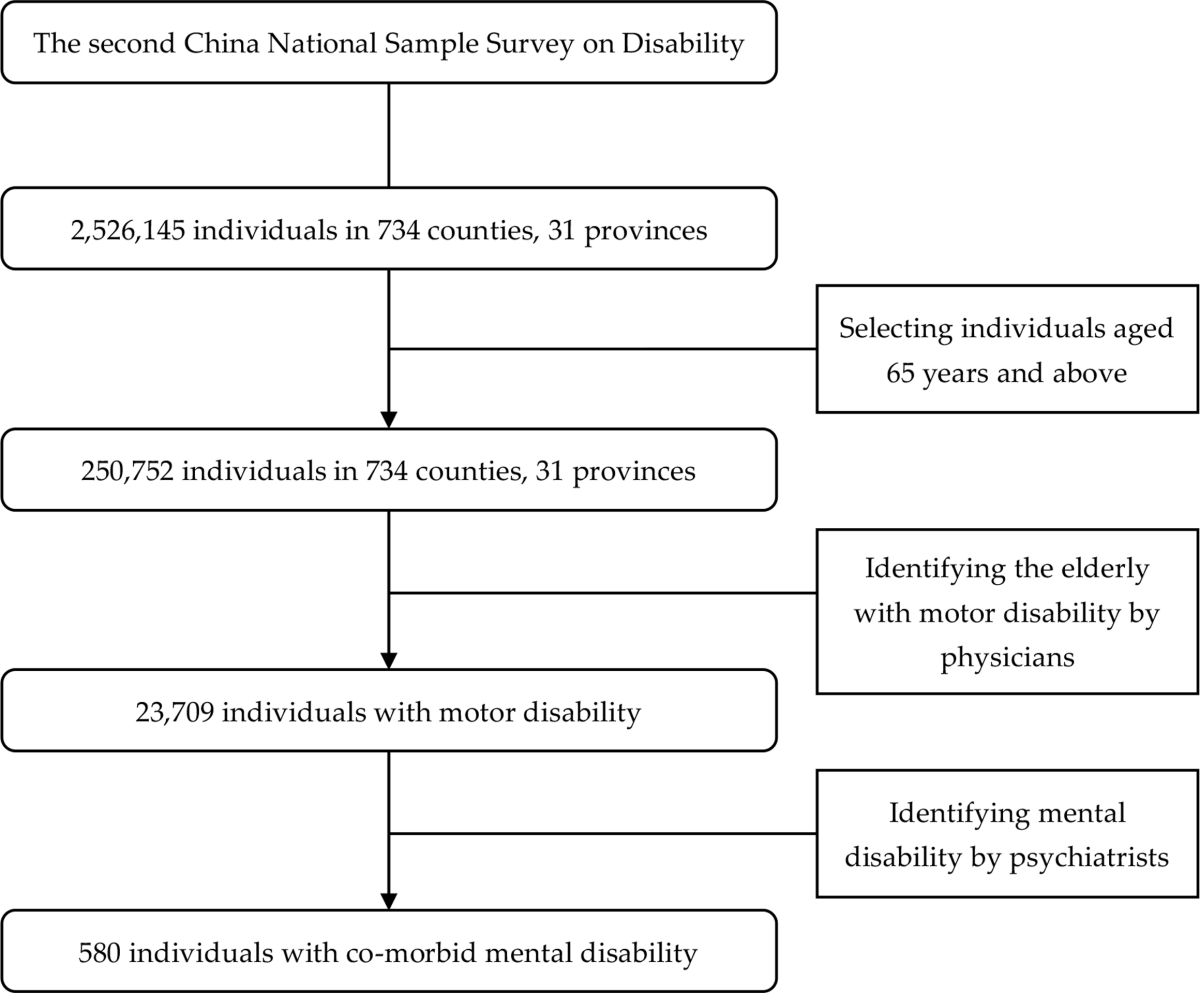
Flowchart of the study samples.

More than 20,000 interviewers, 50,000 survey assistants, as well as 6,000 doctors of various specialties were involved in the survey. In the survey, non-institutional population in all province-level administrative regions of mainland China were sampling population. Within each stratum, a four-stage stratified random cluster sampling with probability proportional to size was used to get the nationally representative samples, following standard procedures for complex samples. Strict quality control measures were implemented at every step during the survey [8]. In total, 2,526,145 persons in 771,797 households were investigated from 5964 sites (arears or communities), 2980 towns (townships or streets), 734 counties (cities or districts) and 31 provinces (autonomous regions or municipalities), representing 1.93 per 1,000 residents of China. In this study, we only considered survey respondents aged 65 years and above, and thus the sample was restricted to 250,752 individuals. More details of the sample selection were shown in Fig 1.

### Measures

In this study, the definition and classification of disabilities were established by the Expert Committee of the CNSSD based on the International Classification of Functioning, Disability, and Health (ICF) [9]. During the survey, each family member of the selected households was interviewed. Trained field interviewers first collected basic household information, after which they used a structured questionnaire to collect information vis-à-vis disabilities. Those who responded “yes” to any of the corresponding questions were referred to different designated physicians for further disability screening and confirmation. Designated physicians performed medical examinations and followed diagnostic manuals to make the final diagnosis, if any, and to confirm its primary causes [10]. Respondents with multiple positive answers were examined by multiple specialists (a separate doctor for each disability). Details about disabilities of the disabled persons with confirmed disability diagnosis were collected by interviewers, and medical and rehabilitation suggestions were also provided to them during the survey.

Motor disability was defined as a loss of motor function of varying degrees or to limitations in movements or activities, resulting from deformed limbs or body paralysis (palsy) or from deformity, caused by damage to the structure or function of those body parts involved in mobility. Those who responded affirmatively to any of the following questions: “Do you or any of your family members has difficulty in walking, standing, sitting, climbing upstairs, holding, writing, washing or dressing by hand in daily life?” were further examined by designed physicians for confirmation of a disability. The medical history, standing, walking, the mutilation or abnormality, movement and function of the trunk and extremities, myodynamia and muscular tension, and movement and abnormality of joints were examined for diagnoses.

Mental disability was defined as any psychiatric condition of over 1-year duration, manifesting as a cognitive, affective, or behavioral disorder and affecting the daily life and social participation of the patient. Those who responded affirmatively to any of the following questions: “Are you or any of your family members forgetful? Do you have difficulty concentrating? Are you unable to control your moods? Are you subject to strange or out-of-the-ordinary behavior? Is there addiction to alcohol or drugs?” were further examined by psychiatrists for confirmation of a disability. Psychiatric disorders, defined according to the International Statistical Classification of Diseases 10th Revision (ICD-10), were diagnosed by psychiatrists. For purposes of this study, co-morbid mental disability among those with motor disability were documented if a certified, motor disabled individual was diagnosed as also having a mental disability, caused by any psychiatric disorder at the time of survey.

The elderly in our study were defined as persons aged 65 years and above. Ages were further categorized as 65–69, 70–74, 75–79 years or >80 years. Survey respondents were also categorized by gender (male or female), minority (yes or no), educational level (illiterate or educated), marital status (married or single), area of residence (rural or urban), annual household income per capita (below or above national average), region (east, central or west), presence of any other disabilities (including visual impairment, hearing loss, speech disability or intellectual disability, yes or no), severity of motor disability (mild, moderate, severe or extremely severe).

### Statistical analysis

Standard weighting procedures for constructing sample weights that allowed for complex survey sample designs were used [11]. Descriptive statistics were used for population-weighted numbers as well as prevalence of co-morbid mental disability among those with motor disability by different characteristics. The Chi-square test was used to examine differences between the proportion of persons having mental disability among those with and without motor disability. The Taylor series linearization method was used to estimate standard errors of proportions for cross-tabulation tables, allowing for both first-stage cluster and stratum variance, and corresponding 99% confidence interval (CI). Univariate logistic regression and multivariate logistic regression were used to calculate the unadjusted and adjusted odds ratios in the association between related factors and mental disability among individuals with motor disability. SURVEYFREQ, SURVEYLOGISTIC, and version 9.1 SAS packages (SAS Institute, Inc., Cary NC, USA) were used for data analyses. For the large survey size, a two-sided p-value < 0.01 was set as statistical significant.

## Results

### Characteristics of the elderly with motor disability

The survey comprised 250,752 Chinese elderly persons. This number is equivalent to a weighted total of 130,033,417 persons. Among them, 23,709 elderly persons (weighted 12,082,371 persons) had motor disability, accounting for ~9.3% of the total number of elderly persons. 24.7% of the elderly with motor disability were the oldest-old >80 years-of-age. Gender-wise, 54.0% of subjects were women. Other demographical, socioeconomic, and disability characteristics were shown in Table 1.

**Table 1:** Characteristics of the elderly with motor disability and the co-morbid mental disability among them.

Variables	Sample No.	Weighted No. (%)	Co-morbid mental disability		
			Weighted No.	Prevalence % (99% CI)	*P*
Age group					0.05
65–69 years-of-age	6016	3053992 (25.28)	60861	1.99 (1.49–2.50)	
70–74	6531	3276801 (27.12)	66267	2.02 (1.51–2.53)	
75–80	5416	2763931 (22.88)	67351	2.44 (1.85–3.03)	
>80	5746	2987647 (24.73)	81128	2.72 (2.09–3.34)	
Sex					0.01
Male	10935	5560452 (46.02)	111255	2.00 (1.62–2.39)	
Female	12774	6521918 (53.98)	164352	2.52 (2.09–2.95)	
National minority					0.74
Yes	2396	1078089 (8.92)	25977	2.41 (1.34–3.48)	
No	21313	11004282 (91.08)	249630	2.27 (1.95–2.59)	
Educational level					0.99
Educated	9923	4840693 (40.06)	110484	2.28 (1.84–2.73)	
Illiterate	13786	7241678 (59.94)	165123	2.28 (1.89–2.67)	
Marital status					0.44
Married	12903	6502837 (53.82)	143669	2.21 (1.83–2.59)	
Single	10806	5579533 (46.18)	131938	2.36 (1.94–2.79)	
Residence					<0.001
Urban areas	7619	3363323 (27.84)	101325	3.01 (2.42–3.61)	
Rural areas	16090	8719047 (72.16)	174282	2.00 (1.64–2.36)	
Province region					<0.001
East region	10229	5099439 (42.21)	167395	3.28 (2.71–3.85)	
Central	6735	3837573 (31.76)	45103	1.18 (0.78–1.57)	
West region	6745	3145359 (26.03)	63109	2.01 (1.46–2.56)	
Household income					0.007
Below national average	20243	10465626 (86.62)	225490	2.15 (1.83–2.47)	
Above national average	3466	1616744 (13.38)	50118	3.10 (2.24–3.96)	
Having any other disabilities^a^					<0.001
No	17322	8813343 (72.94)	84421	0.96 (0.75–1.67)	
Yes	6387	3269027 (27.06)	191187	5.85 (4.92–6.77)	
Severity of motor disability					<0.001
Mild	12543	6427875 (53.20)	80341	1.25 (0.97–1.53)	
Moderate	5832	2941116 (24.34)	68473	2.33 (1.73–2.93)	
Severe	3671	1872423 (15.50)	72923	3.89 (2.95–4.84)	
Extremely severe	1663	840957 (6.96)	53870	6.41 (4.74–8.07)	

a. including visual impairment, hearing loss, speech disability or intellectual disability

### Co-morbid mental disability among motor disability

Among the elderly with motor disability, a weighted number of 275,607 persons (2.28%, 99% CI: 1.97–2.59) had co-morbid mental disability, which was significantly higher than those without motor disability (1.01%, P<0.001). In multivariable analysis, the elderly with motor disability were 2.11 times (99% CI: 1.83–2.43) more likely to be co-morbid mental disabled than those without motor disability (p<0.001; Table 2).

**Table 2:** The association between mental disability and motor disability among Chinese elderly.

Independent variable	Co-morbid mental disability			Univariate analysis^a^		Multivariable analysis^a^^,^^b^	
	Sample No.	Weighted No. (%)	*P*	OR (99% CI)	*P*	OR (99% CI)	*P*
			<0.001		<0.001		<0.001
Not having motor disability	23129	1193310 (1.01)		reference		reference	
Having motor disability	580	275607 (2.28)		2.28 (1.98–2.64)		2.11 (1.83–2.43)	

a. The dependent variable was whether having co-morbid mental disability or not (reference = not) and the independent variable was whether having motor disability or not (reference = not).

b. Controlling for age, sex, nationality, educational level, marital status, household income, residence, region, and having any other disabilities or not.

Organic mental disorder was the leading cause of co-morbid mental disability, with a prevalence of 1.87% (99% CI: 1.59–2.15), followed by schizophrenia, schizotypal or delusional disorders (0.26%, 99% CI: 0.17–0.36). Other cause-specific prevalence of co-morbid mental disability was shown in Table 3.

**Table 3:** Cause-specific co-morbid mental disability among the elderly with motor disability.

Cause-specific co-morbid mental disability	Sample No.	Weighted No.	Prevalence (%)	99% CI
Any mental disorder	580	275607	2.28	1.97–2.59
Organic mental disorders	484	226058	1.87	1.59–2.15
Mental disorders due to psychoactive substance use	4	2223	0.02	0.00–0.04
Schizophrenia, schizotypal or delusional disorders	59	31878	0.26	0.17–0.36
Mood disorders	12	6021	0.05	0.01–0.09
Neurotic stress-related and somatoform disorders	9	3123	0.03	0.01–0.05
Other causes	12	6305	0.05	0.01–0.09

### Factors of co-morbid mental disability among the elderly with motor disability

The overall prevalence of co-morbid mental disability was significantly higher among the older ones, females, urban residents, persons living in the east or west regions, those in households with higher income, those with other co-morbid disabilities, and those with more severe motor disability (Table 1).

In multivariable analysis, urban areas (OR: 1.38; 99% CI: 1.04–1.84), east (2.60, 1.76–3.83) or west (1.65, 1.06–2.55) regions, having other co-morbid disabilities (5.93, 4.48–7.85), and having more severe motor disability (Moderate: 1.81, 1.28–2.57; Severe: 2.90, 2.08–4.04; Extremely severe: 4.22, 2.96–6.01) were significantly associated with higher risk of co-morbid mental disability among the elderly with motor disability (Table 4).

**Table 4:** The factors associated with the co-morbid mental disability among the elderly with motor disability.

Variables	Univariate analysis			Multivariable analysis		
	OR	99% CI	*P*	Adjusted OR	99% CI	*P*
Age group			0.04			0.16
65–69 years-of-age	reference			reference		
70–74	1.02	0.72–1.45		0.88	0.61–1.25	
75–80	1.23	0.87–1.73		0.86	0.60–1.23	
>80	1.37	0.97–1.94		0.72	0.49–1.05	
Sex			0.01			0.07
Male	reference			reference		
Female	1.27	0.99–1.62		1.23	0.92–1.66	
National minority			0.74			0.13
Yes	reference			reference		
No	1.06	0.66–1.71		1.32	0.83–1.12	
Educational level			0.99			0.73
Educated	reference			reference		
Illiterate	1.00	0.78–1.29		1.04	0.76–1.43	
Marital status			0.44			0.77
Married	reference			reference		
Single	1.07	0.85–1.35		0.97	0.75–1.26	
Residence			<0.001			0.004
Rural areas	reference			reference		
Urban areas	1.52	1.16–2.00		1.38	1.04–1.84	
Province region			<0.001			<0.001
Central	reference			reference		
East region	2.85	1.95–4.19		2.60	1.76–3.83	
West region	1.72	1.11–2.67		1.65	1.06–2.55	
Household income			0.002			0.02
Below national average	reference			reference		
Above national average	1.45	1.06–1.99		1.34	0.97–1.86	
Having any other disabilities^a^			<0.001			<0.001
No	reference			reference		
Yes	6.42	4.89–8.43		5.93	4.48–7.85	
Severity of motor disability			<0.001			<0.001
Mild	reference			reference		
Moderate	1.88	1.34–2.64		1.81	1.28–2.57	
Severe	3.20	2.30–4.45		2.90	2.08–4.04	
Extremely severe	5.41	3.84–7.62		4.22	2.96–6.01	

a. including visual impairment, hearing loss, speech disability or intellectual disability

## Discussion

Data for this study were gleaned from representative samples of the most recent national population–based survey on disability in China. Results indicate that a significant number of elderly Chinese persons (>65 years-of-age) were living with co-morbid mental and motor disabilities, i.e. ~2.28% of the physical disabled persons. A national survey in Singapore adults indicated that the prevalence of comorbid mental and medical disorders was 6.1% in the adult resident population [12]. The lower figure in our research is reasonable considering a frequently lower prevalence in disabilities compared to disorders with lower severity.

In previous studies, a compelling relationship has been observed between the presence of motor disabilities and the risk for mental disorders [13,14]. For example, two previous studies in China reported that the prevalence of depression symptom was significant higher in persons with motor disability than those without motor disability [15,16]. Our findings further indicated that the presence of motor disability was also significantly associated with a higher risk of having a co-morbid mental disability. The strong association can be partly explained by the consequent social exclusion, social isolation, lack of social participation, and loneliness, which numerous studies have shown to be strong correlates of various mental disorders [14]. Especially for the elder persons, motor disability could reduce the social and psychological resources the elderly received and felt, such as less contacting with family and friends, and a gradual loss of control and dignity, leading an increased risk of mental disorders [17,18]. Additionally, the decline in outdoor activities and body function loss caused by motor disability, the self- and social neglect in the elderly and inadequate door-to-door mental healthcare services, cause barriers to mental health services use among the elderly with motor disability, which contributed to the development of mental disorders into mental disabilities [19,20].Currently, the Chinese government is developing the Healthy China Plan as a national strategy, in which health promotion for the entire population and throughout the total life cycle are the two main themes, while elders and persons with disability are two of the key populations. Our results highlighted the importance to provide more medical and rehabilitation services for the elderly to prevent the co-morbid physical and mental disabilities among them.

The current study found that having more severe motor disability and other disabilities expect motor disability were associated with an increasing co-morbid mental disability risk. It is consistent with previous studies that indicated the number of difficulties in activities of daily living (ADL) and instrumental activities of daily living (IADL), and the severity of disability were directly related to the likelihood of respondents having depression [14,7].

Notably, our results indicated that except a traditional vulnerable status, i.e. living in the west province region, some higher socioeconomic statuses (SES) such as urban areas and the east region were also associated with a higher risk of a co-morbid mental disability. This seems inconsistent with previous studies on the association between SES and mental disorders or disability in general population, which usually indicated mental disorders were more likely found in persons with lower SES [21]. However, study population in the current study are elderly with motor disability, thus our exceptional findings may reflect some particularities of this kind of elders in China.

For those with higher SES, they had once had a better social participation opportunity, while as the existence of motor disability, they lose their opportunity. Thus, our findings may be partly explained by the big psychological letdown for the those with motor disability in these superior areas or regions. Additionally, as the busy daily life and work in the urban areas and developed regions, the family member of elders with motor disability may have more limited time to take care and support the disabled [22]. A previous study reported a higher prevalence of mental disorders among persons with disabilities caused by injury in urban areas compared to rural areas [23], which supports our findings. Our findings highlight that more targeted strategy was need for the population with essentially higher SES such as living in urban areas or east regions in addition to the traditional vulnerable groups.

This study has several limitations. Firstly, the stringent definition of disability used in the survey was responsible for reporting low prevalence rates of disabilities, thus indicating the importance of employing more international comparable definitions for future studies. Additionally, the cross-sectional design does not provide direct evidence of causality, so that our survey lacked data on clinical/patient outcomes resulting from this co-morbid condition. Based on the fact that the study was based on a large, representative population-based sample covering all provincial areas of China, this study provides a new and broader understanding of co-morbid mental and motor disability, regardless of its limitations.

In conclusion, our findings reported the size and prevalence of older population living with co-morbid mental and motor disability in China. Elderly with motor disability have a high risk of having a co-morbid mental disability, especially those with essentially higher SES. As China is undergoing health reform, these findings highlight the need of and will be beneficial for implementing more comprehensive prevention and rehabilitation strategies, to prevent and control the emergence and development of multiple disabilities and promote the physical and mental health of the elderly.
